# Dried Milk

**Published:** 1905-01-14

**Authors:** 


### EDITOR'S LETTER-BOX.

[Our Correspondents are reminded that prolixity is a great bar to publication, and that brevity of style and conciseness of statement greatly facilitate early insertion.]

DRIED MILK.

The Galak Milk Products, Limited, writes: Sir,—As manufacturers of dried milk, we cannot, in justice to ourselves, allow your article to go unchallenged.

Allow us to reply to your remarks seriatim.

It is claimed for our dried milk that it is superior to ordinary milk inasmuch as every precaution, based on scientific principles, has been used to insure freedom from bacteria and consequent sterility. As we are enabled to get from 2,000 to 4,000 gallons of milk per day, and at a price unheard of by hospital authorities, so we are enabled to offer milk powder at the price identical with ordinary cow's milk. We consider we get a purer milk in the first instance than any ordinary hospital, as our machines are in the midst of the dairy pastures. Take the ordinary deliveries of milk to London hospitals. The milk is very often from six to twelve hours old, before it enters the hospital gates.

The representative you mentioned in your article who stated he had a milk powder containing 27 per cent. of butter fat was quite right, and we rather think the boot is on the other leg about "colossal ignorance." We have never heard about the Government standard of 3 per cent. for milk powder, and we should certainly like to get hold of a Jersey cow which would lactate milk powder. We do claim that a dry substance like milk powder is more likely to be germ free than liquid milk, and as "Galak" is consumed a short time after reconstitution, there are not the same facilities for contamination as in the case where ordinary milk has to travel scores of miles and pass several handlings.

We can supply a milk powder containing as much as 30 per cent. of butter fat—and one which if diluted to the strength of 1 in 8 would show an average 3·7 per cent. of cream fats and this percentage always normal.

In the course of a long letter Mr. James R. Hatmaker states:—As a reader of The Hospital I am surprised to see the article on "Milk Powders" that appeared in your issue of December 31st. It is so inaccurate that it would appear to be malicious.

The milk-powder referred to in your article as containing 1·25 per cent. of fat was evidently milk-powder obtained by drying separated milk. But the representative who said he could supply a milk-powder containing 27 per cent. of cream (butter fat) was right, for when the water is evaporated from full-cream milk the dry powder resulting has 27 per cent., or a little more of fat. The Government standard to which you refer is for liquid milk. When the water is evaporated the percentage of fat increases, as you can easily understand.

I am prepared to verify the statements I have made herein.

. *. It is hardly a satisfactory explanation to say that a milk powder can be obtained with a sufficient proportion of fat, when the sample first submitted has been proved to be deficient in this constituent.—Ed. The Hospital.

